# Fabrication and Biocompatibility of Electrospun Silk Biocomposites

**DOI:** 10.3390/membranes1040275

**Published:** 2011-10-10

**Authors:** Kai Wei, Byoung-Suhk Kim, Ick-Soo Kim

**Affiliations:** Nano Fusion Technology Research Group, Faculty of Textile Science & Technology, Shinshu University, Ueda, Nagano 386-8567, Japan; E-Mail: sudaweikai@hotmail.com

**Keywords:** silk, nanofiber, fibroblast, tetramethoxysilane, hydroxyapatite, osteoblast

## Abstract

Silk fibroin has attracted great interest in tissue engineering because of its outstanding biocompatibility, biodegradability and minimal inflammatory reaction. In this study, two kinds of biocomposites based on regenerated silk fibroin are fabricated by electrospinning and post-treatment processes, respectively. Firstly, regenerated silk fibroin/tetramethoxysilane (TMOS) hybrid nanofibers with high hydrophilicity are prepared, which is superior for fibroblast attachment. The electrospinning process causes adjacent fibers to ‘weld’ at contact points, which can be proved by scanning electron microscope (SEM). The water contact angle of silk/tetramethoxysilane (TMOS) composites shows a sharper decrease than pure regenerated silk fibroin nanofiber, which has a great effect on the early stage of cell attachment behavior. Secondly, a novel tissue engineering scaffold material based on electrospun silk fibroin/nano-hydroxyapatite (nHA) biocomposites is prepared by means of an effective calcium and phosphate (Ca–P) alternate soaking method. nHA is successfully produced on regenerated silk fibroin nanofiber within several min without any pre-treatments. The osteoblastic activities of this novel nanofibrous biocomposites are also investigated by employing osteoblastic-like MC3T3-E1 cell line. The cell functionality such as alkaline phosphatase (ALP) activity is ameliorated on mineralized silk nanofibers. All these results indicate that this silk/nHA biocomposite scaffold material may be a promising biomaterial for bone tissue engineering.

## 1. Introduction

Continuous progress in surgical technologies and biomedical science has allowed tissue or whole-organ transplantation to become potential options to restore native functions such as regeneration of fractured or diseased bones. Unfortunately, the increasing demand for function transplants and the human aspiration for longer living far exceed the available supply of usable donor tissues. Transplantation technology has encountered severe limitations. Therefore, new technologies are needed to reduce this gap in clinical need *versus* available healthy tissue and organ supplies. In recent years, electrospinning has been employed as a leading technique for generating biomimetic scaffolds made of synthetic and natural polymers for tissue engineering. This method can produce electrospun fibers with diameters in the range from several micrometers down to less than 100 nm that have a very high surface area to mass ratio. This kind of thee dimensional, fibrous scaffold with high porosity can closely biomimic native extracellular matrix, facilitate cell attachment, support cell growth, and regulate cell differentiation [1,2].

Silk filament derived from silkworm *Bombyx mori* is a natural protein mainly made of sericin (the outer coating) and fibroin (the inner brins). The sericin protein is removed by a process called degumming in industry, so that the term silk is commonly improperly used to define only one of its two components, the silk fibroin. Silk fibroin is a typical fibrous protein that has been studied as a scaffold for tissue engineering because of its excellent biocompatibility, bioabsorbability and low level of inflammatory potential [3,4,5]. In recent years, regenerated silk fibroin nanofibers have been developed using electrospinning technique for tissue engineering [4,5].

In tissue engineering *in vitro*, many researches were directed towards the development of novel hybrid nanofibers scaffold using regenerated silk fibroin by electrospinning technique [6,7,8,9] in order to improve cell adhesion, proliferation and differentiation behaviors. In current research, various electrospun nanofibers have been devised to prepare biomimetic nanocomposites for potential application in tissue engineering. For instance, Mather *et al.* prepared silica from nanofibers by immersion of the PEI/PVP (poly(ethylene imine)/poly(vinyl pyrrolidone)) nanofibers in silica precursor tetramethylorthosilicate (TMOS) and then calcinations [10]. A simple alternative to create silk/silica composites is to coat silk-based material templates with silica precursors (such as tetraethylorthosilicate (TEOS)) and subsequently heat them at 105 °C for several hours, as was demonstrated with cocoon fibers of *Bombyx mori* fibroin silkworms [11]. Furthermore, the silk template can subsequently be removed by calcinations, yielding a porous material in which the pore structure is determined by the silk-based material.

Bioactive ceramic such as hydroxyapatite (HA) has also been used in many medical applications in orthopedic and dental surgery owing to its osteoconductivity and osteophilicity [12,13,14]. In the past few years, various electrospun nanocomposite fibers, such as PCL/CaCO_3_ [15], HA/gelatin [16], silk/HA [17], PLA/HA [18], and triphasic HA/collagen/PCL [19] have been devised and explored for potential bone regeneration applications. However, since most of these electrospun composite fibers were prepared by electrospinning of blends made by simply mixing the prior obtained inorganic nanoparticles with viscous polymer solutions, it usually results in composite nanofibers with very limited or lacking of specific interactions between the organic and inorganic phases [20].

In this study, two kinds of silk-based composite nanofibers were prepared by electrospinning for improving cell cultivation. Firstly, we describe the formation of regenerated silk fibroin/tetramethoxysilane (TMOS) nanofibers obtained by electrospinning of their blends. Afterwards, hydrolysis and condensation reactions of alkoxy silicon monomer (TMOS) produce the porous network structures composed of the Si–O–Si bonds. Moreover, the amine groups catalyze the hydrolysis process due to the alternating presence of protonated and non-protonated amine groups in the fibroin molecular chains, which allows hydrogen bond formation with the oxygen adjacent to silicon in the precursor and thus facilitates –Si–O–Si– bond formation [21]. Here we hypothesize that the hybrid of silk fibroin and TMOS could improve hydrophilicity of the fibrous nanocomposites; furthermore, it would improve cell activity by accelerating adhesion behavior in the early stages. Secondly, a silk-nHA (nano-hydroxyapatite) biocomposite scaffold was also developed by an electrospinning technique and then post-treated by employing a calcium phosphate (Ca–P) alternate soaking method. We hypothesize that well-distributed HA nanoparticles on the silk nanofibrous biocomposites would improve cell activity by accelerating differentiation in the late stages. Extensive material characterizations and cell culture studies using MC3T3-E1 are conducted to assess the viability and potential application of this material for future bone graft applications.

## 2. Materials and Experiments

### 2.1. Materials

Silkworm Bombyx mori is a natural protein that is mainly made of sericin (the outer coating) and fibroin (the inner brins). The sericin protein is removed by a process called degumming in industry, so that the term silk is commonly improperly used to define only one of its two components. In this work, the cocoons of Bombyx mori were degummed three times in an aqueous Na_2_CO_3_ (0.02 M) at 100 °C for 30 min and washed with distilled water in order to remove sericin from the surface of silk fibers and then the silk fibroin was obtained. The silk fibroin was then dissolved in a ternary solvent system of CaCl_2_/CH_3_CH_2_OH/H_2_O in 1:2:8 molar ratios at 70 °C with vigorous stirring. After dialysis against distilled water with cellulose tubular membrane with molecular weight cutoffs (MWCO) ranging from 12,000 to 16,000 Daltons for 4 days at 25 °C, the regenerated silk fibroin sponge was obtained by lyophilization (−20 °C, 24 h).

### 2.2. Electrospinning

In the electrospinning system [22,23], a high-voltage power supply (Har-100*12, Matsusada Co., Tokyo, Japan), capable of generating voltages up to 100 kV, was used as the source of the electric field. The regenerated silk protein solution was contained in a plastic tube connected with a capillary tip with an inner diameter of 0.6 mm. The copper wire connected to a positive electrode (anode) was inserted into the polymer solution, and a negative electrode (cathode) was attached to a metallic collector. The solution volume was controlled to keep proper flow rate for spinning.

For silk/TMOS nanofiber, the solutions were prepared by dissolving 8% (w/w) regenerated silk protein in 1,1,1,3,3,3-Hexafluoro-2-propanol (HFIP), after 24 h stirring, 5% and 15% (on the weight of silk fibroin) of TMOS was added to the fibroin solution within 30 mins under stirring [24,25,26]. For silk/nHA nanofiber, the silk fibroin solutions in the concentration of 18% (w/w) were prepared by dissolving the regenerated silk protein sponge into 98% formic acid, and used for electrospinning [27]. The collecting roller was placed at a distance of 10 cm from the capillary tip and a voltage of 16 kV was applied to the wire in the capillary using a high voltage power supply (MATSUSADA Precision Inc., Kusatsu-City, Japan), while the receiving roller was rotating. All the processes were carried out at room temperature.

### 2.3. Mineralization

Mineralization of silk nanofibers was performed using a Calcium–Phosphate (Ca–P) alternate soaking method. That is, mineralization of nHA was achieved by subjecting the nanofibers in a series of calcium and phosphate treatments, deemed as the alternate soaking method [28]. Silk nanofibrous scaffolds were first immersed in 0.5 M of CaCl_2_ (pH of 7.2) (Aldrich Chemical Company, Inc., St. Louis, MO, USA), followed by rinsing with deionized (DI) water. Afterwards, the scaffolds were subsequently immersed in 0.3 M of Na_2_HPO_4_ (pH of 8.96) (Merck and Co. Inc., Whitehouse Station, NJ, USA) and rinsed with DI water. The above-mentioned step was referred to as 1 cycle of Ca–P treatment. All nanofibers were subjected to 3 cycles of Ca–P treatments, where the first cycle was 10 min (in each chemical solution) and the second and third cycles were 5 min (in each chemical solution). After mineralization, the nanofibers were freeze-dried overnight. nHA residues, which were not adhered to the silk nanofibers, were also collected in the DI water for X-ray diffraction (XRD) analyses.

### 2.4. Cell Culture

The silk fibroin nanofibers and its composite nanofibers (collected on round cover glass slips of 15 mm in diameter) were immersed in 80% ethanol for 2 h for sterilization purposes. The nanofibers were washed with phosphate buffered saline (PBS) thrice followed by culture medium thrice to eliminate any residual ethanol. MC3T3-E1 osteoblast-like cells, which were obtained from the RIKEN Cell Bank (Tsukuba, Japan), were cultured until passage 7 and seeded on the silk fibroin nanofibers and its composite nanofibers at a cell concentration of 2 × 10^4^ cells/well. Cells were incubated at 37 °C, in a 5% CO_2_ atmosphere incubator, using α-modified minimal essential medium (α-MEM; GIBCO). The medium comprised of 10% heat-inactivated fetal bovine serum (FBS), 100 U/mL penicillin, 100 U/mL streptomycin and 0.1% β-glycerophosphate was used to induce osteoblastic differentiation. For all cell investigations, cells cultured on TCDs (tissue culture dishes, high-grade polystyrene Nunc™ Dishes, Thermo Fisher Scientific, Roskilde Site, Denmark) were evaluated as controls. The medium was changed every two days to ensure that there was an adequate supply of nutrients present in the culture plate.

### 2.5. Cytotoxicity Assay and Live/Dead Cell Staining

Cell live/dead staining was performed to determine the number of viable and non-viable L929 cells, using a Cellstain-Double Staining Kit (Dojindo Laboratories, Kumamoto, Japan). After 12 h cultivation on the nanofibrous scaffolds, L929 cells were stained with PBS containing 2 μM Calcein AM and 4 μM propidium iodide (PI) for 15 min at 37 °C, according to the previous method we used [23,29]. Calcein AM reacts with intracellular esterase to produce green fluorescence at 490 nm, while PI enters only dead cells with damaged membranes to produce red fluorescence at 545 nm, upon binding to nucleic acids. Digital images of viable (green) and dead (red) cells in selected areas were visualized using a Zeiss Axio Imager M1 fluorescence microscope, equipped with AxioCam MRm (Carl Zeiss MicroImaging GmbH, Munich, Germany).

### 2.6. Immunocytofluorescence Staining for Nuclei, Vinculin, and Filamentous Actin

After seeding and 24 h cultivation as per section cell culture, the nanofibrous scaffolds were treated with a continual fluorescent staining of Alexa Fluor 568 (vinculin, red fluorescence at 568 nm), fluorescein phalloidin (filamentous actin, green fluorescence at 490 nm), and 4′,6-diamidino-2-phenylindole (nuclei, blue fluorescence at 345 nm) as previously reported [29]. Staining was visualized using a Confocal Laser Scanning Microscope (FLUOVIEWFV1000D, OLYMPUS, Tokyo, Japan) equipped with Olympus IX81.

### 2.7. Proliferation Assays

Proliferation assays were determined by MTT method after incubation of cells on the nanofibrous scaffolds for 1, 3, 5, 7, 10 and 14 days. The MTT ([3-(4,5-dimethylthiazol-2-yl)-2,5-diphenyltetrazolium bromide]) assay measures the mitochondrial (metabolic) activity on the cell culture, which reflects the viable cell number. The cultures were washed with PBS. Then 100 μL of MTT solution was added to each culture well followed by 3 h incubation in 5% CO_2_ at 37 °C. MTT solution is consisted of 0.5% (w/v) MTT/PBS solution: RPMI1640 culture medium = 1:9 (v/v). 100 μL of acid isopropanol (0.1 N HCl in anhydrous isopropanol) was added to all wells and mixed thoroughly to dissolve the purple MTT formazan crystals (yielding from mitochondrial dehydrogenases of viable cells which cleave the tetrazolium ring). The resulting purple solution is spectrophotometrically measured by absorbance at 595 nm using a Biotrack II plate reader (GE Healthcare, Amersham Place, Little Chalfont, England). The data reported were the mean value of 3 examinations.

### 2.8. Cell Attachment

Cells were seeded and cultured as under the same conditions discussed in cell culture section. Cells in culture medium were counted (Nm) after 30, 60 and 90 min of incubation. The cell adhesion ratio for each condition was calculated using the following equation: Adhesion ratio (%) = (1 − Nm/2 × 10^4^) × 100. All data reported were the mean of thee examinations.

### 2.9. Alkaline Phosphatase (ALP) Activity

The level of cell differentiation on the nanofibrous scaffolds was assessed by determining the level of ALP activity. ALP activity was measured at time points of 1, 3, 5, 7, 10 and 14 days. 500 μL of β-nitrophenyl phosphate solution containing 1 mM MgCl_2_ (Aldrich Chemical Company, Inc., St. Louis, MO, USA) was added to the medium and incubated for 10 min at 37 °C. The enzymatic reaction was stopped by adding 500 μL of 0.2 N NaOH. Finally, the absorbance was recorded at 405 nm.

### 2.10. Statistical Analysis

The data collected were expressed as mean ± standard deviation (SD). The two-tailed Student's T-test (T-test) was employed to obtain p values, enabling determination of the level of significance of the data. P-values of less than 0.05 (p < 0.05) were considered to be of significant difference.

### 2.11. Characterization

The morphology of electrospun silk composite nanofibers was observed with scanning electron microscopy (SEM, S-3000N, Hitachi Co., Tokyo, Japan). The average fiber diameter of the scaffolds was determined from 50 measurements of the random fibers using an image analysis software (Image J, National Institutes of Health, Bethesda, Maryland, USA). Fourier Transform Infrared (FTIR) spectrophotometer (IR Prestige-21, SHIMADZU, Kyoto, Japan) using the standard KBr disk method was employed to determine the types and molecular interactions of functional groups presenting in these hybrid nanofibers. The spectra were recorded from 500 to 4,000 cm^−1^ at a resolution of 4 cm^−1^. The thermal stability was carried out with a TG/DTA apparatus (TG/DTA6200, Seiko Instruments Inc., Tokyo, Japan) by heating from 40 °C to 600 °C under a continuous nitrogen purge of 20 mL/min. The heating rate was 10 °C/min. Advancing water contact angle experiments were performed by using a contact angle goniometer (FACE. CA-D, Kyowa Interface Science, Chiba, Japan). The droplet is deposited by a syringe pointed vertically down onto the sample surface, and a high resolution camera captures the image, which can then be analyzed either by eye or using image analysis software. The crystallographic phases of the nHA were analyzed using a powder X-ray diffraction (XRD) diffractometer (Rotaflex RTP300, Rigaku Co., Tokyo, Japan; 40 kV, 150 mA). All XRD measurements were carried out using Cu Kα1 radiation, at a wavelength of 1.5402 Å with a scanning speed of 2.0°/min and a sampling interval of 0.02°. The 2θ range was fixed at 10–60°.

## 3. Results and Discussion

### 3.1. Fabrication of Silk/TMOS Composite Nanofibers

#### 3.1.1. Morphology of Silk/TMOS Nanocomposites Scaffolds

**Figure 1:**
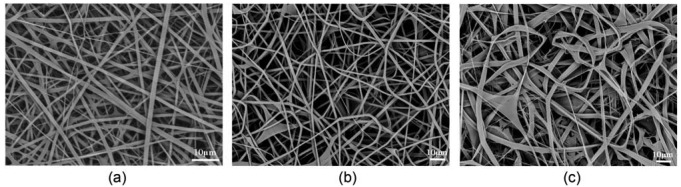
Scanning electron microscope (SEM) images of the nanofibers (**a**) regenerated silk fibroin nanofibers; (**b**) silk/tetramethoxysilane TMOS hybrid nanofibers with TMOS concentration of 5 wt %; and (**c**) silk/TMOS hybrid nanofibers with TMOS concentration of 15 wt %.

Figure 1(a) shows SEM image of pure regenerated silk fibroin nanofibers electrospun from a regenerated silk solution dissolved in HFIP at a concentration of 8 wt %. SEM analysis indicates a broad diameter distribution, with an average diameter of 1,250 nm and standard deviation (SD) of 410 nm.

The silk/TMOS nanofibers, shown in Figure 1(b,c), were obtained by adding 5 wt % TMOS in 8 wt % regenerated silk fibroin solution within 30 min under stirring, electrospinning at a voltage of 16 kV and a TCD of 10 cm, and finally drying at 25 °C for 24 h under humidity of 20%. Interestingly, the adjacent fibers in silk/TMOS hybrid electrospun nanofibers caused to ‘weld’ at fiber contact points [30], as confirmed by SEM images (Figure 1(b)). Compared to welded hybrid fibers, the pure silk nanofibers shown in Figure 1(a) were intact and did not show flash welding. Additionally, the fiber diameters showed almost the same size of 1,290 nm (SD = 370 nm) (Figure 1(b)) as the pure silk nanofibers (Figure 1(a)). The observed ‘weld’ at contact points may be due to the equilibrium water content, as was verified by thermogravimetric analysis (TGA) analysis shown later. Moreover, as the TMOS concentration increased to 15%, the fibers became belts and the juncture extended like a sheet which could not be identified as previously reported nanofiber mats (Figure 1(c)). So in this study we investigated the hybrid nanofibers with the TMOS concentration of 5%.

#### 3.1.2. Secondary Structure of Electrospun Regenerated Silk/TMOS Nanofibers

**Figure 2:**
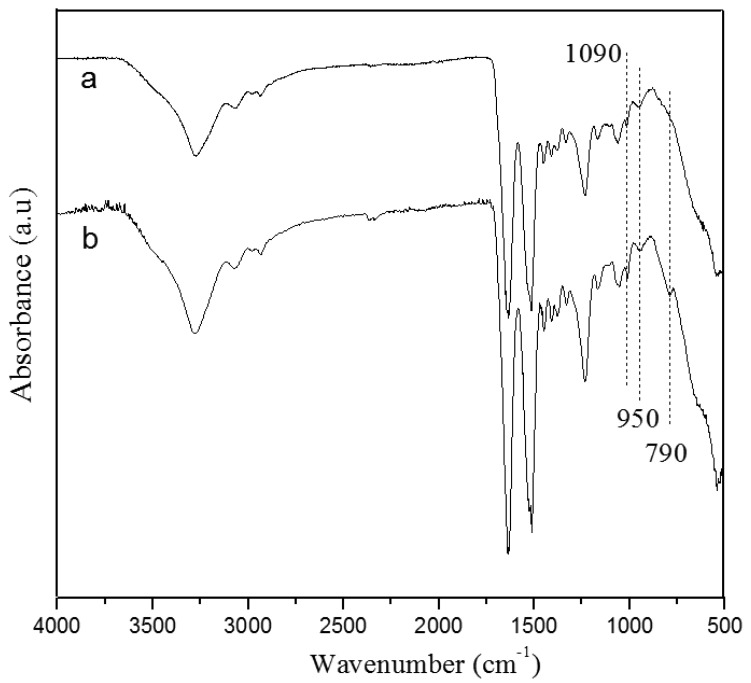
Fourier Transform Infrared (FTIR) spectra of: (**a**) pure silk nanofibers; and **(b**) silk/TMOS nanofibers with TMOS concentration of 5 wt %.

FTIR spectra of electrospun pure silk nanofibers and resultant silk/TMOS nanofibers were shown in Figure 2. Three important absorbance peaks: –Si–OH stretching at 950 cm^−1^, –Si–O–Si– symmetric stretching at 790 cm^−1^ and –Si–O– asymmetric stretching at 1,090 cm^−1^ (each position was indicated by vertical dotted lines in Figure 2) were observed. The formation of siloxane group in the electrospun fibers was clearly evident from the –Si–O–Si– vibration band at 790 cm^−1^, which is otherwise absent in the spectra of pure silk nanofibers. Furthermore, a slight increase in intensity at 1,090 cm^−1^ and 950 cm^−1^ indicating the Si–O and Si–OH stretching vibrations appeared in the hybrid nanofibers. The increase in intensity of the broad peak around 3,400 cm^−1^ assigned to –OH groups, showed either the silanol group or the –OH group in the water or alcohol liberated due to the condensation reactions of TMOS. Indeed, the observed –OH peak occurred despite extensive drying of silicified fibers for 24 h at 25 °C in vacuum. Peaks corresponding to various groups in silk fibroin were also presented in the region of 1,200–2,000 cm^−1^, indicating very little or no change in pure silk and silk/TMOS nanofibers.

#### 3.1.3. Thermal Properties of Electrospun Regenerated Silk/TMOS Nanofibers

**Figure 3:**
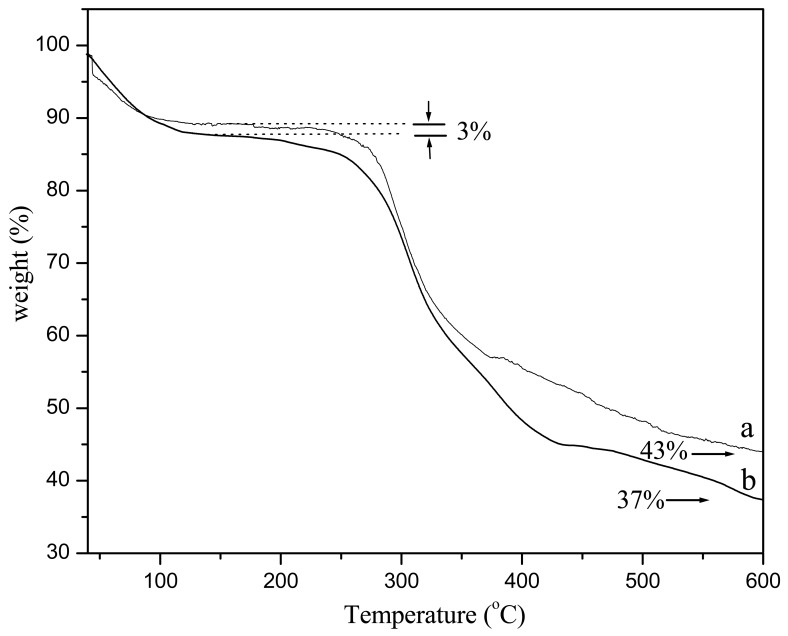
TGA curves of (**a**) pure silk nanofibers; and (**b**) silk/TMOS nanofibers with TMOS concentration of 5 wt %.

TGA experiments enabled further quantification of water content in hybrid silk/TOMS nanofibers. We should note that other sources of moisture may exist and are not directly controlled in our experiments, such as trace moisture in TMOS, as well as moisture from the air during electrospinning. Figure 3 revealed that the silk/TMOS nanofibers had an inorganic content around 37 wt % at 600 °C which was lower than pure silk nanofiber. We reason that the silk/TMOS nanocomposite has higher equilibrium moisture content. As evident from Figure 3, a 10% weight loss of the silk nanofibers occurred at temperatures below 120 °C, roughly indicating the level of water absorption by the fibers. TGA characterization allowed us to estimate the water content in the silk/TMOS fibers. As a result, the present study revealed that the addition of TMOS resulted in higher water content in the resultant silk/TMOS fibers.

#### 3.1.4. Hydrophilicity Properties of Electrospun Regenerated Silk/TMOS Nanofibers

**Figure 4:**
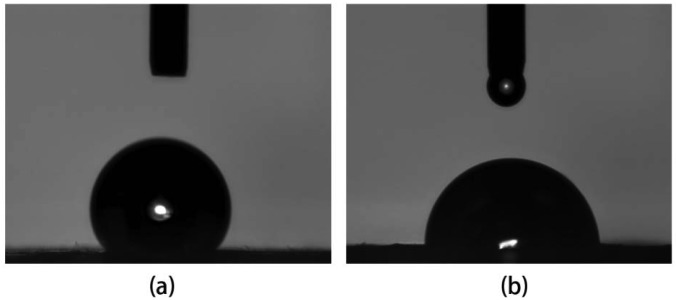
Water contact angle of (**a**) pure silk nanofibers; and (**b**) silk/TMOS nanofibers.

The hydrophilicity of electrospun nanofiber composites can be seen from Figure 4. Water contact angle showed a sharp decrease of electrospun silk nanofibers incorporated with TMOS than pure regenerated silk fibroin nanofiber from 116.2° to 84.8°. Although silk fibroin has many hydrophilic groups such as –OH and –COOH, the incorporation of TMOS results in higher water capacity in the resultant fibers due to the formation of spatial net structures formed via Si–O–Si– linkages as proved by TG result. Furthermore, the water contact value of silk/TMOS hybrid nanocomposites was closed to that of TCD (Tissue culture Dish) template (75.6°), which indicated that it may be more suitable for cell attachment than pure silk nanofibers because the optimum water contact angle of the surface for fibroblast adhesion is reported in the range between 55° and 75° [31]. The cell adhesion behavior will be discussed in the following text.

#### 3.1.5. Cytotoxicity Assay

**Figure 5:**
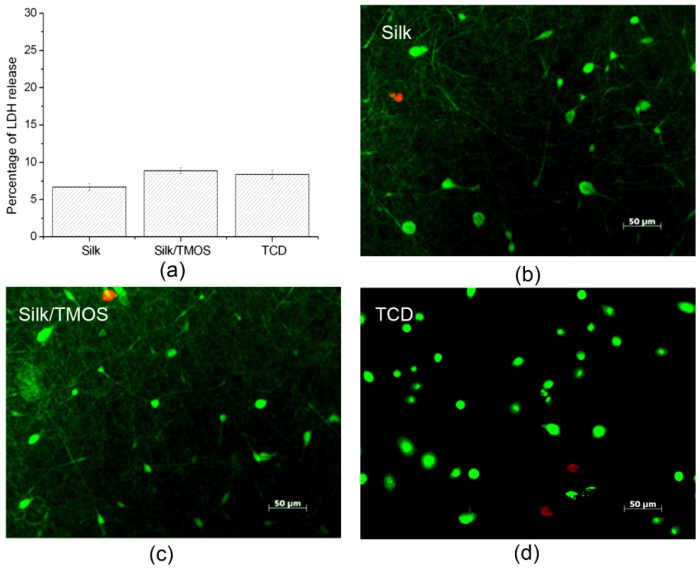
Lactate dehydrogenase (LDH) release (**a**) and fluorescence micrographs of Calcein AM/PI-stained L929 cells with live cells fluorescing green and dead cells fluorescing red after 12 h culture on the Silk/TMOS; (**b**) silk; (**c**) nanofibrous scaffold; and TCD (**d**).

Lactate dehydrogenase (LDH) leakage assay results shown in Figure 5(a) suggest that cell culturing on silk/TMOS fibrous scaffolds cause LDH release near 8% while that on silk is near 7%. Both of them showed no significant difference in the LDH release, compared to TCD as a control. The results indicated that the incorporation of TMOS in the fibrous material did not affect the excellent biocompatibility of silk fibroin. From the live/dead fluorescence micrographs in Figure 5(b,c), the majority of cells incubated for 12 h on silk/TMOS and silk scaffolds were alive and parts of them revealed spindle shaped morphology. Cytotoxicity assays indicate that L929 cells on silk/TMOS fibrous scaffold have comparable viability on silk fibrous scaffold.

#### 3.1.6. Adhesion Behavior of Electrospun Regenerated Silk/TMOS Nanofibers

**Figure 6:**
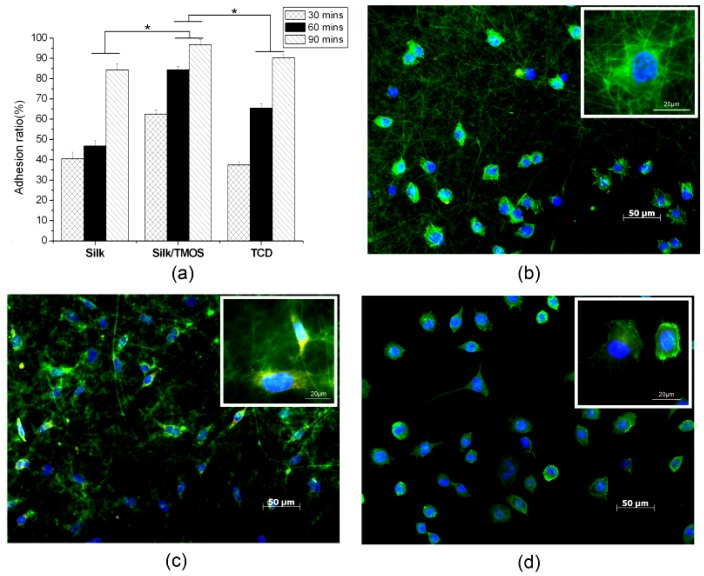
The adhesion ratio for L929 cells after 90 min culture on pure silk, silk/TMOS nanofibers and tissue culture dishes (TCD) controls (**a**). Significant difference between different materials groups were denoted as * (p < 0.05). Fluorescent staining of F-actin (green), vinculin (red), and cell nuclei (blue) for L929 cells after 6 h culture on silk: fibrous scaffold **(b**), silk/TMOS (**c**), and TCD (**d**).

The adhesion ratio of L929 cells on pure silk, silk/TMOS nanofibers and TCD controls are shown in Figure 6(a). The cell adhesion ratio of silk/TMOS nanofibers was significantly higher than pure silk nanofibers and TCD controls in all the culture times, it reached as high as 95% after 90 min' cultivation while that on pure silk was near 85%. Although an increase in adhesion ratio on both pure silk nanofibers and TCD controls after 30 to 90 min of cell culture were observed, adhesion ratio of silk/TMOS nanofibers showed excellent attachment behavior to L929 cells which could be attributed to the melioration of hydrophilicity. The incorporation of TMOS on silk fibroin nanofibers had enhanced the adhesion behavior of L929 cells as expected.

Immunofluorescence microscopy of L929 cells grown on pure silk, silk/TMOS fibrous scaffold and TCD after 6 h cultivation are shown in Figure 6(b–d). Blue fluorescence of cell nuclei revealed round, well-spaced, and regularly distributed nuclei across the surface of both fibrous scaffolds. Compared to the L929 cells (round shaped morphology) on pure silk, the cytoskeletal organization (green fluorescence) of most cells on silk/TMOS scaffold showed obvious spindle-shaped morphology, while both round and spindle-shaped L929 cells have been investigated on TCD as a control. Moreover, only L929 cells on silk/TMOS showed vinculin signals (red fluorescence) at the extremities of cellular extensions. Consistent with the adhesion ratio in Figure 6(a), these results reveal a better adhesion and stretching behavior for L929 cells on silk/TMOS nanofibrous scaffolds than on a pure silk scaffold.

Accordingly, intensive researches have been carried out in order to manipulate cellular behavior by modifying the relative properties of materials. M. Kuzuya *et al.* induced durable hydrophilicity on the hydrophobic of polystyrene surface and further modified it by Arg-Gly-Asp (RGD) sequence which can be recognized by the receptor protein on the cellular membrane to enhance the adhesion and proliferation of PC12 cell [32]. M. Lensen *et al.* induced stable cell adhesion by manipulating the surface topography to the hydrogel poly (ethylene glycol) although fibroblast is intrinsically non-adhesive to the smooth surface [33]. M. Hamdan *et al.* found that the positive surface of the titanium cylinder resulted in favorable NCTC clone 929 fibroblast cell adhesion [34]. The results in our study suggested that the cell adhesion ratio and spreading on silk/TMOS have been enhanced compared to the pure silk. This can be explained by the change of fibrous surface properties in terms of hydrophilicity and surface morphology change. First of all, water contact angle showed that silk/TMOS had better hydrophilicity than neat silk because of the formation of spatial net structures formed via Si–O–Si– linkages. Studies about the wettability, initial protein adsorption, and the cell adhesion showed that one of the fibronectin states had more active conformation (via secondary structure rearrangements) being on a hydrophilic surface [35,36]. This will consequently lead to more spreading of fibroblasts and ultimately sufficient cell adhesion and spreading. It has been reported that the optimum wettability of the surface for fibroblast adhesion is in the range between 55° and 75° [31]. The TCD used in this study as control showed a water contact of 75.6° (data not shown) while the incorporation of TMOS has changed to the relatively hydrophobic silk surface from 116.2° to 84.8°. Secondly, SEM images in Figure 1(b,c) showed the interesting adjacent fibers in silk/TMOS hybrid electrospun nanofibers caused by ‘weld’ morphology at contact points. It has been known that the substrate's topography has a great influence on the behavior of cells at interface. It showed that contact guidance happened to cells of different types on different materials with different sizes and shapes of patterns [37,38,39]. Probably, this kind of ‘weld’ morphology in silk/TMOS nanofibrous mats influence the surface microstructure of the fiber that might has positive effect to the L929 cell adhesion, although more intensive study is necessary for the conclusion. Nevertheless, considering the complexity of cell surface interaction, which involves protein absorption and specific binding, the function groups that existed in TMOS and net charges presented on the silk/TMOS hybrid scaffolds might also influence the protein adsorption and therefore cell adhesion to some degree [40,41].

### 3.2. Fabrication of Silk/nHA Composite Nanofibers

#### 3.2.1. Morphology of Silk/nHA Nanofibrous Scaffolds

**Figure 7:**
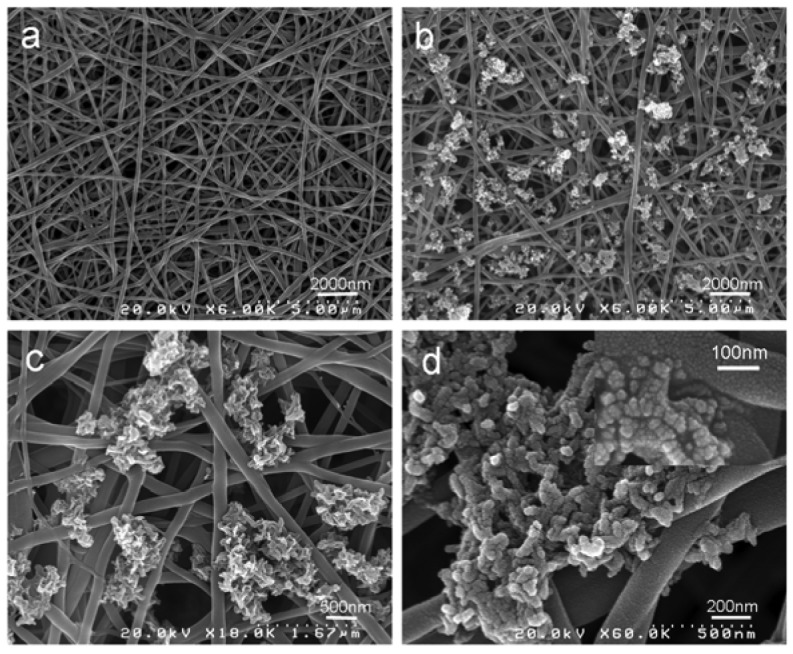
FE-SEM images of pure silk and mineralized silk/nano-hydroxyapatite (nHA) nanofibers after 3 cycles of Ca–P treatment. (**a**) pure silk nanofibers (6,000×; scale bar, 500 nm); (**b–d**) mineralized silk/nHA nanofibers after 3 cycles of Ca–P treatment with different magnification.

Mineralization of nHA was successfully deposited on pure silk fibroin nanofibers after 3 cycles [42] of Ca–P treatment as depicted in Figure 7. As shown in Figure 7(b,c), the diameter of obtained silk fibroin nanofibers was around 242 ± 34 nm. It was observed that nHA was homogenously formed on pure silk nanofiber substrates. As evidenced in the high resolution FE-SEM micrograph (Figure 7(d)), nHA particles formed on silk fibroin nanofibrous scaffolds were nanocrystalline in structure and the deposition occurred predominately on the surfaces of the nanofibrous scaffolds. The size of nHA particles was approximately 30–35 nm in diameter, which was proved by XRD analysis (see below).

#### 3.2.2. Crystal Structure of Silk/nHA Nanofibrous Scaffolds

**Figure 8:**
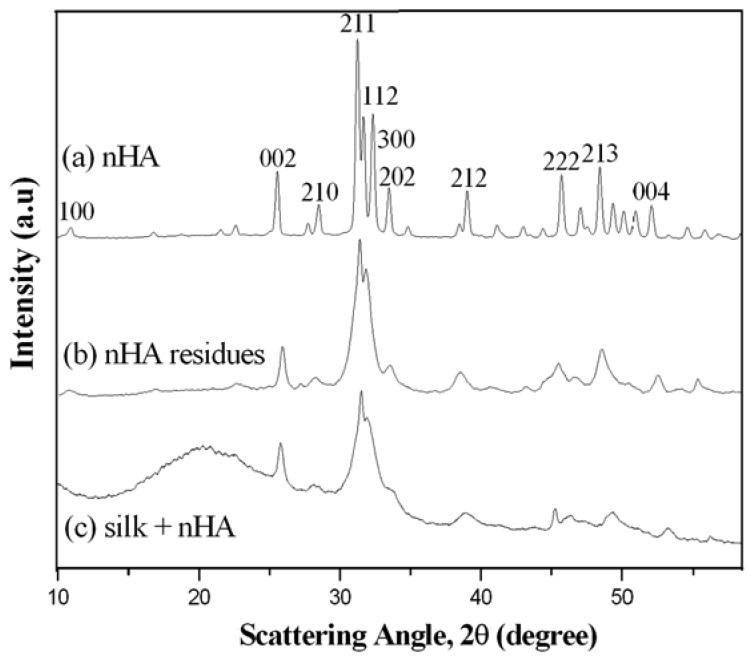
X-ray diffraction (XRD) patterns of (**a**) pure hydroxyapatite (HA) (control); (**b**) nHA residues; and (**c**) mineralized silk/nHA nanofibers.

XRD results clearly demonstrated the presence of nHA in the mineralized silk/nHA nanofibrous scaffolds (Figure 8(b): nHA residues and Figure 8(c): mineralized silk/nHA nanofibers). The broad halo peak at 20.6° in Figure 8(c) was attributed to the silk II form of β-sheet crystalline structure [43,44]. All the peaks in Figure 8(b,c) were consistent with the peaks associated with pure nHA (Figure 8(a)), suggesting that rapid mineralization approach used in our study was effective in producing nHA phases on the silk nanofibrous substrates. Unfortunately, both the Energy Disperse X-ray Analysis (EDX) and XRD analyses indicate the poor crystallinity of the nHA formed on silk nanofibers. It can be explained that the hydroxyl or amide group, which existed in the silk fibroin macro chains, captured calcium or phosphate ions in the solution by chelation. The supply of calcium or phosphate ions to the apatite nuclei was retarded, and the apatite crystals grew under the condition that calcium or phosphate ions were not sufficiently supplied. Therefore, the crystal growth of apatite was inhibited along a particular axis and resulted in random orientations of crystals in the mineralized fibroin [45].

**Figure 9:**
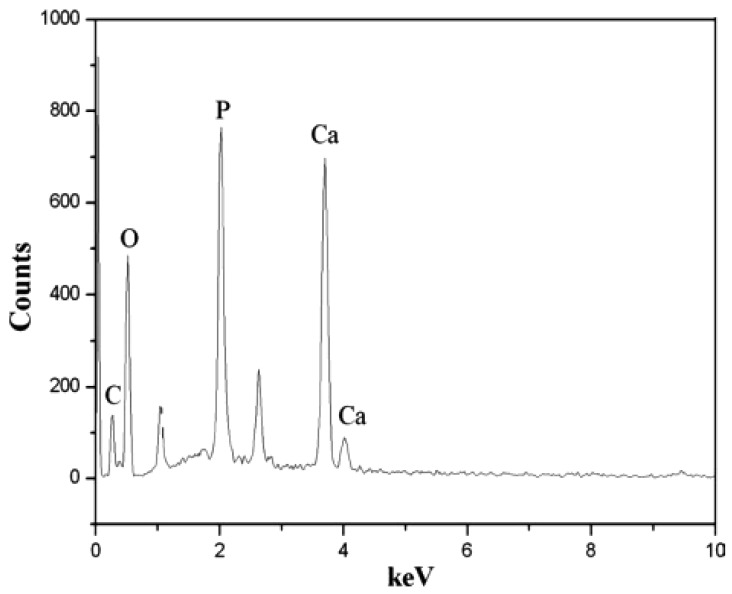
Energy Disperse X-ray Analysis (EDX) patterns of mineralized silk/nHA nanofibers.

Figure 9 shows EDX (Energy Disperse X-ray Analysis) patterns of mineralized silk/nHA nanofibers. It was found that the Ca/P atomic ratio in the nHA prepared in this work was lower than 1.67 of theoretical atomic ratio in the pure HA, which might be indicative of the presence of Ca-deficient apatites. In addition, the full width at half-maximum (FWHM) of the strongest characteristic peak (211) was used to estimate the average crystallite size by applying the following Debye–Scherrer equation, D = κλ/βcosθ, where X-ray wavelength λ was 1.5402, k the shape factor which was often assigned a value of 0.89 if the shape was unknown, D the average diameter of the crystals in angstroms, θ the Bragg angle in degrees, and β the full width at half-maximum of the strongest characteristic peak in radians. It was found that the average crystallite size of pure nHA was 30 nm while both nHA residues and nHA nanoparticles deposited on the silk nanofibers were *ca.* 32 nm, which also agreed well with FE-SEM result, as shown in Figure 7(d).

#### 3.2.3. Secondary Structure of Silk/nHA Nanofibrous Scaffolds

**Figure 10:**
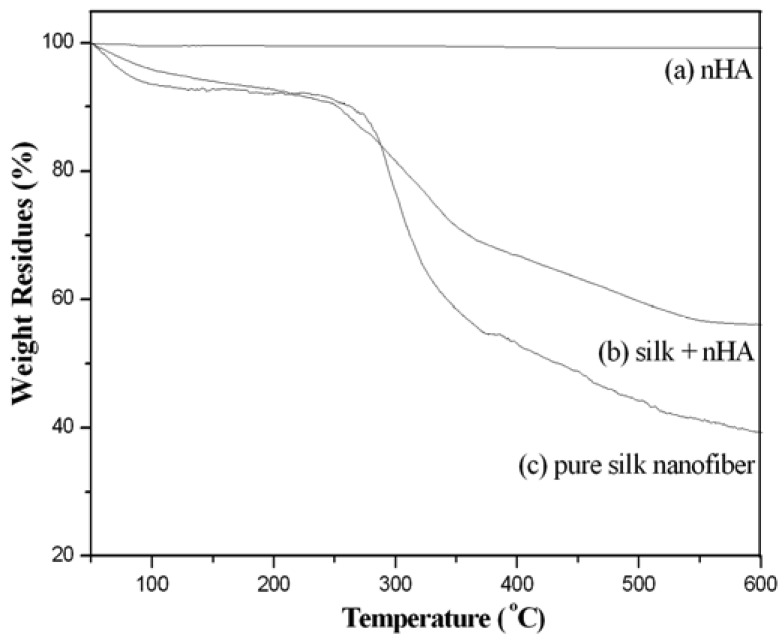
FT-IR spectra of (**a**) pure HA (control); (**b**) pure silk nanofibers; and (**c**) mineralized silk/nHA nanofibers.

FT-IR spectrum was carried out to confirm the functional groups in the mineralized silk/nHA nanofibrous scaffolds. Our observations demonstrated that both phosphate and carbonate groups were presented in mineralized silk fibroin nanofibers (Figure 10(c)), suggesting the successful mineralization by a Calcium–Phosphate (Ca–P) alternate soaking method. The O–P–O bending mode was characterized at approximately 559 cm^−1^ and 603 cm^−1^. The bands centered at 1,010 cm^−1^ and 959 cm^−1^ corresponded to the P–O stretching vibration modes, which were caused by the PO_4_^3−^ group in HA [46,47]. For carbonate groups, the peak was observed at around 873 cm^−1^ which was ascribable to C-O stretching vibration mode on CO_3_^2−^ group [48]. Furthermore, the C=O stretch vibration of amide I (1,625 cm^−1^) and N–H bending modes of amide II (1,529 cm^−1^), which were characteristic bands for silk fibroin, were also observed in the IR spectra as seen in Figure 9(b,c). However, when the spectra of mineralized silk fibroin/nHA composites was compared with that of pure silk fibroin nanofibers, the intensity of the two amide peaks of fibroin/nHA composites decreased severely, because of the formation of the bonding between Ca ions and C=O bonds. It has been reported that in the initial stage of collagen mineralization, collagen prefers to chelate calcium ions in solution and these chelate ions subsequently form nucleation sites of calcium phosphate crystals [49]. The oxygen atoms of carboxyl groups and carbonyl groups on the surface of fibroin molecules were incorporated with calcium ions and then served as the nucleation sites for apatite formation and, consequently, the nHA crystals were precipitated on the surfaces of silk fibroin nanofibers.

#### 3.2.4. Thermal Properties of Silk/nHA Nanofibrous Scaffolds

**Figure 11:**
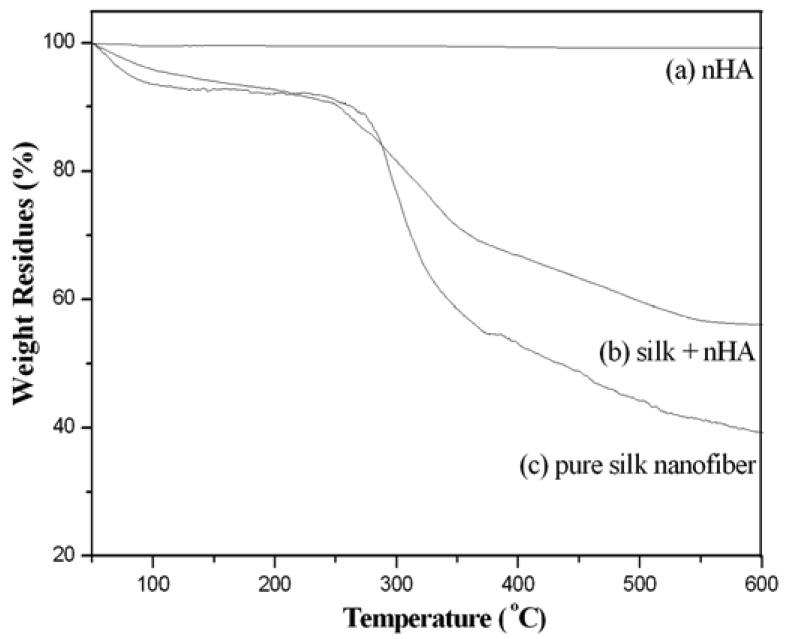
(**a**) TGA curves of pure nHA; (**b**) mineralized silk/nHA nanofibers; and (**c**) pure silk nanofibers.

From our TG results, there was no significant weight loss of pure nHA when the temperature was raised from 50 °C to 600 °C (Figure 11(a)). A weight loss of about 7% around 90–105 °C was detected in pure silk fibroin nanofibers, which was ascribed to the evaporation of water. As the temperature is increased further, the weight residue started to decrease sharply at 295–305 °C (Figure 11(c)) due to the thermal degradation of silk protein [50]. At 600 °C, the weight residue was about 28.5%. The similar water content (about 5%) in the mineralized silk fibroin/nHA composite scaffolds was also observed around 90–105 °C (Figure 11(b)). Moreover, similar thermal degradation behavior with a dramatic decrease in weight residue was observed at around 300 °C. About 48.4% residue was remained at 600 °C for the mineralized silk/nHA composite scaffolds. It was therefore found that the amount of nHA in mineralized silk nanofibers was *ca.* 20%.

#### 3.2.5. Proliferation Behavior of Silk/nHA Nanofibrous Scaffolds

**Figure 12:**
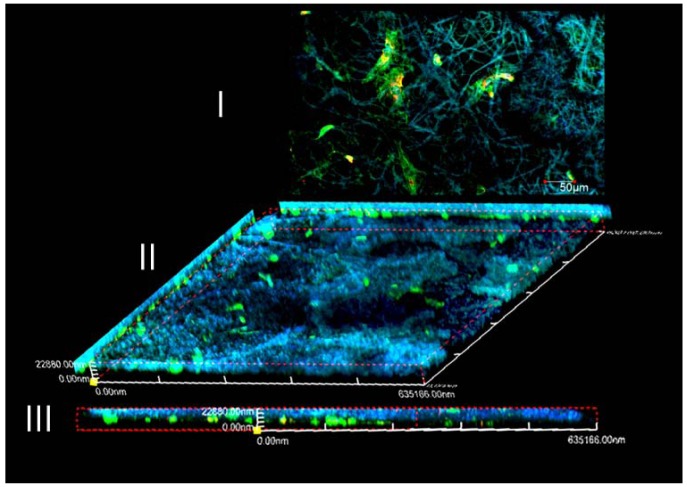
Fluorescent staining of F-actin (green), vinculin (red), and cell nuclei (blue) for MC3T3-E1 cells after 24 h cultivation on silk/nHA fibrous scaffold. (**I**) 2D morphology of cultivation; (**II**) 3D morphology by laser scanning of fibrous scaffold; and (**III**) cross section of II.

In Figure 12, immunofluorescence of actin filaments demonstrates the cytoskeletal organization (green). Since the high surface area to volume of nanofibers which is used to mimic the extracellular matrix environment of cells, the MC3T3-E1 cells in Figure 12(I) is investigated to spread in spindle or polygonal morphology after 24 h cultivation. Moreover, intensive vinculin signals can be found along the stretching cellular axis. The MC3T3-E1 cell's adhesion activity in Figure 12(I) suggested that the mineralization of nHA on silk fibrous mats did not outweigh the benefit of silk nanofibrous scaffold. 3D network culturing morphology of MC3T3-E1 in Figure 12(II) was determined by laser depth-of-focus scanning about 20μm of the silk-nHA scaffold. Together with the cross-section image of Figure 12(III) where a portion of cells are penetrated into fabricated channels, silk-nHA fibrous mats were proved to be suitable for supporting MC3T3-E1's 3D cultivation.

**Figure 13:**
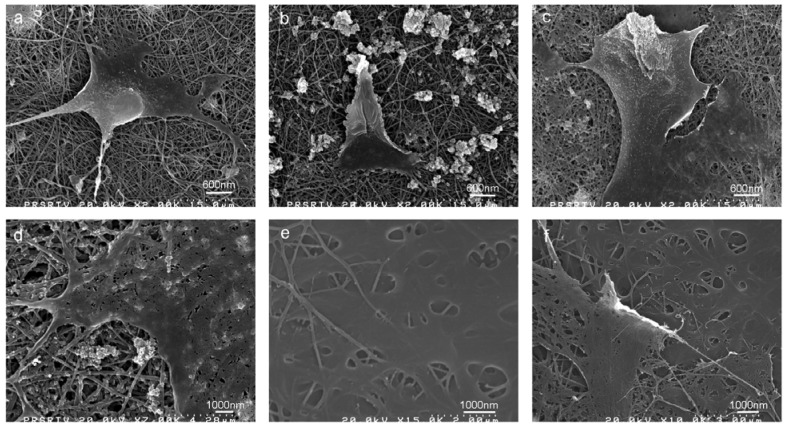
Osteoblasts on pure and mineralized silk/nHA nanofibers. (**a**) pure silk (day 1); (**b**) silk/nHA (day 1); (**c**) pure silk (day 2); (**d**) silk/nHA (day 2); (**e**) pure silk (day 7); and (**f**) silk/nHA (day 7).

As evidenced in the FE-SEM micrographs, osteoblasts were successfully seeded on both pure and mineralized silk nanofibers where the cells were partly adhered to nHA regions in the silk/nHA nanofibers (Figure 13(b)). The deposition of nHA did not affect the MC3T3-E1 attachment compared to those grown on pure silk nanofibers after 1 day cultivation (Figure 13(a,b)) [51,52]. Likewise, cell spreading in a spindle-like shape was also observed on HA-based composites after 2 days of cell culture due to the physical contacts between cells which is maintained via the formation of filopodia or lamellipodia [53]. As seen in Figure 13(d), the cells were strongly anchored on the silk/nHA nanofibrous scaffolds, with preferential attachment of the pseudopodia to nHA regions. In addition, a greater cell spreading on silk/nHA nanofibers was observed after 2 days of cell culture (Figure 13(d)), compared to that after 1 day (Figure 13(b)). When the culture period is prolonged in our study, full cell coverage was found on the nanofibers, and eventually osteoblasts covered most of the nanofiber surfaces after 7 days of cell culture with extended lamellipodia, creating a cell multi-layers on the fibers (Figure 13(f)).

**Figure 14:**
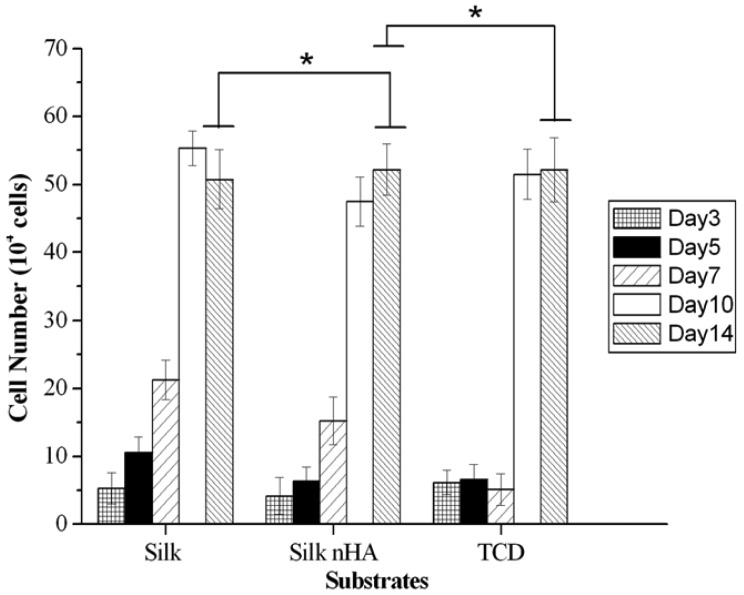
Cell proliferation on pure and mineralized silk nanofibers after 3 to 14 days of cell culture. Significant difference between different materials groups were denoted as * (*p* ≥ 0.05).

Figure 14 shows cell proliferation on pure and mineralized silk nanofibers after 3 days of cultivation. It was observed that when compared with the pure silk nanofibers, the cell numbers were smaller for mineralized silk/nHA nanofiber scaffold and TCD until 7 days cultivation. This is different from what was observed in other studies where osteoblast proliferation was improved on nanophase HA materials [54,55]. Probably, the difference was due to the size effect of hydroxyapatite nanoparticles on proliferation as well as the density or bulk distribution. Moreover, previous studies showed that curved nHA surface at a nanometer level could decrease osteoblast-like cells on early period of proliferation [56]. The previously reported results [57,58,59] also coincided with those observed in our study: surface topography had a crucial influence on cell behavior, which was accompanied by attenuated proliferation rates on rougher surfaces. Nevertheless, after 14 days of cultivation, cell number on mineralized silk is of no significant differences between pure silk and TCD controls (*p* ≥ 0.05), suggesting that the addition of nHA had no negative effect on later period of cell proliferation.

#### 3.2.6. Alkaline phosphatase (ALP) Activity of Silk/nHA Nanofibrous Scaffolds

**Figure 15:**
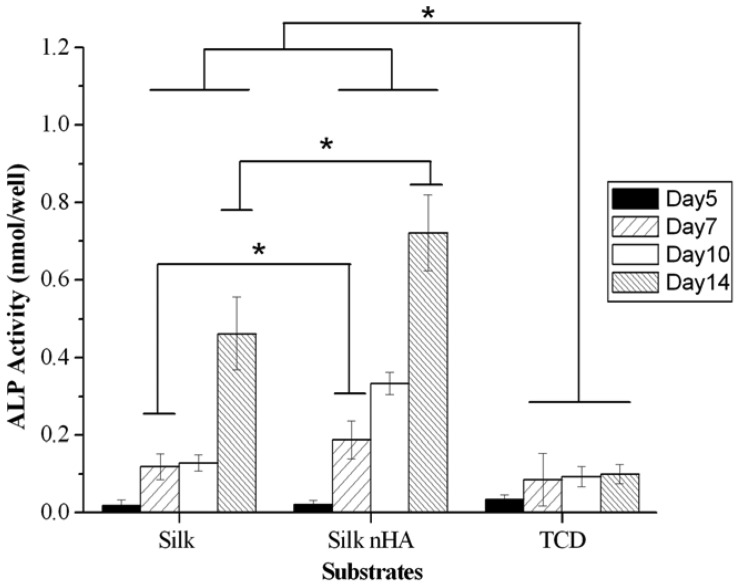
Alkaline phosphatase (ALP) activity on pure and mineralized silk/nHA nanofibers after 5, 7, 10 and 14 days of cell culture. Significant difference between different materials groups were denoted as * (p < 0.05).

One of the properties of nHA is its bioactive nature which promotes osteoblastic differentiation *in vitro* [60,61,62]. ALP-hydrolyzed phosphate esters play an essential role in the initiation of the cell differentiation process. Thus, ALP activity, as a marker of osteoblastic activity and a standard to evaluate the differentiation of MC3TC-E1 cells, were measured and shown in Figure 15. There was a slight reduction in ALP activity on the pure silk and mineralized silk/nHA nanofibers than TCD after 5 days of cell culture, while a significant increase in ALP activity on both pure silk and mineralized silk/nHA nanofibers after 7 to 14 days of cell culture, compared to TCD counterparts. Results of ALP activity of pure silk and mineralized silk/nHA nanofibers were comparable to an early stage after 5 days of cell culture, but after 7 days of cell culture ALP activity was meliorated in mineralized silk/nHA than pure silk substrates. The incorporation of nHA on silk fibroin nanofibers had enhanced the differentiation activity of MC3T3-E1 from day 7 to 14. After 14 days of cell culture, ALP activity on mineralized silk/nHA nanofibers was nearly 1.6 times higher than that of pure silk nanofibers. One noteworthy observation was that ALP activity in pure silk and mineralized silk/nHA nanofibers was superior to that of TCD as a control from 7 to 14 days of cell culture.

## 4. Conclusions

We have successfully prepared two different nanofibrous biocomposites based on regenerated silk fibroin via electrospinning and post-treatment processes. Regenerated silk fibroin/TMOS hybrid nanofibers showed superior fibroblast attachment, compared to pure silk fibroin nanofibers, due to relatively higher hydrophilicity. Accordingly, the silk/TMOS nanofibrous composites showed a sharp decrease in water contact angle than pure regenerated silk fibroin nanofiber due to the spatial net structure formed via –Si–O–Si– connections which were responsible for water capacity. Interestingly, the electrospinning process caused adjacent fibers to ‘weld’ at contact points, as confirmed by SEM analysis. This study of simple incorporation of silk with TMOS has merit for preserving the excellent biocompatibility of silk, and the fibrous three-dimensional silk/TMOS scaffold can support significantly enhanced L929 adhesion more effectively than pure silk. Thus this method might open a new pathway to prepare various functional scaffolds with enhanced bioactivity for *in vitro* tissue engineering application.

Furthermore, nano-hydroxyapatite was successfully deposited on regenerated silk fibroin nanofibrous scaffolds by a biomimetic Ca–P method. It was found that the primary nHA crystals with a diameter of about 30 nm were well distributed on the surface of the nanofibrous substrates. The ALP expression of the cells was improved on mineralized silk/nHA nanofibers during the cell culture periods, irrespective of the cell number which was leveling off (3 to 7 days). It appeared that the nHA presenting in mineralized silk/nHA nanofibers had a greater improvement effect on differentiation stages than in the early stages of cultivation, such as adhesion and proliferation. The cell cultivation in this study demonstrated that silk/nHA nanocomposite scaffold could support the early stage of osteoblast adhesion and had a significant effect on the differentiation stage, suggesting that this composite scaffold may be a promising biomaterial for bone tissue engineering.
